# Health and well-being of the Portuguese citizens: impacts of the COVID-19

**DOI:** 10.1186/s41687-023-00628-1

**Published:** 2023-09-05

**Authors:** Lara N. Ferreira, Luís N. Pereira, Pedro L. Ferreira

**Affiliations:** 1 Universidade do Algarve, Faro, Portugal; 2 Research Centre for Tourism, Sustainability and Well-Being (CinTurs), Faro, Portugal; 3 Centre for Health Studies and Research of the University of Coimbra (CEISUC), Coimbra, Portugal; 4 Faculty of Economics, University of Coimbra, Coimbra, Portugal

**Keywords:** COVID-19, Wellbeing, Quality of life, Social inclusion

## Abstract

**Background:**

COVID-19 pandemic placed unprecedented pressure on societies and healthcare systems around the world. Over the last years, measures imposed in almost all countries dealing with the pandemic sent the entire world into an extensive crisis and thus into a deep global recession. Since the outbreak began, many European countries have faced three/four waves of pandemic. Portugal has mainly dealt with three waves (March/April’2020; October/November’2020; January/February’2021), the third being the deadliest one. The purpose of this article is to provide evidence on the impact of the COVID-19 on health-related quality of life (HRQol) and well-being (W-B) of Portuguese citizens. It aims to (i) characterize these outcomes during the COVID-19 pandemic; (ii) compare them to pre-COVID-19 Portuguese population; and (iii) identify the social determinants that may affect these outcomes during the COVID-19 pandemic.

**Methods:**

This study used data from a survey that collected data on HRQoL, W-B, satisfaction with life, economic and labour impacts, access to healthcare, mental and physical health, amongst others. The survey was implemented by telephone to a representative random sample of 1,255 respondents from the general adult Portuguese population, stratified by sex, age group and region. Data was collected during the end of the second national lockdown. For comparison purposes, we have also used two other representative databases from the general Portuguese population: (i) data from before the pandemic (n = 1,006); and (ii) data from a survey conducted during the first lockdown, (n = 904).

**Results:**

Looking at health and access to healthcare, 4% of citizens had their surgeries postponed or cancelled because of COVID-19, more than a quarter had medical appointments or complementary exams postponed or cancelled, with 7% over 65 years old with surgeries cancelled or postponed and 32% medical appointments. COVID-19 pandemic also impacted negatively on the HRQoL of citizens, especially in the first lockdown. Half of the respondents reported feeling nervous, anxious, or on edge, about 45% of citizens felt sad or depressed. Sleeping problems were reported for almost 39% of citizens, and loneliness is reported by 29% of citizens. For about 70–85% of citizens, these feelings were more so than before the pandemic. Citizens with fair/strong economic stability were the most economically affected by the pandemic.

**Conclusions:**

We provided evidence on the impact of COVID-19 on health and W-B of Portuguese citizens. Their health was worse than before the pandemic and the access to healthcare was highly affected.

**Supplementary Information:**

The online version contains supplementary material available at 10.1186/s41687-023-00628-1.

## Background

COVID-19 pandemic placed unprecedented pressure on societies and healthcare systems around the world. Over the last two years, measures imposed in almost all countries dealing with the pandemic led the entire world into an extensive crisis and thus into a deep global recession.

Since the outbreak began, many European countries have faced six or seven waves of COVID-19 pandemic. Portugal has dealt with six waves (March/April’2020; October/November’2020; January/February’2021; July/August’2021; December’2021/February’2022; May/June’2022), the third being the deadliest one.

In the first wave, as all over the world, Portuguese government realised that National Health Service (NHS) was unprepared to deal with the new emerging infectious disease. It focused on prevention of cases by designing national public health measures of home isolation and quarantine, with serious economic consequences, but also reduced access to healthcare for non-COVID-19 patients (see [1–2] for a thorough list of measures). In the second wave, more localized measures were adopted, such as confinement applied to particular counties or cities, according to the number of cases and risk of infection. While the Portuguese authorities, following World Health Organization’s recommendations, tried to delay national measures of home confinement, by the end of January it became clear to everyone that, if no strict and hard measures of a national confinement were taken, Portuguese NHS would collapse. Indeed, in third wave, the number of deaths and new cases increased, and Portugal was classified as the worst European country dealing with pandemic. Therefore, by the end of January 2021, another emergency state was declared, and strict measures were imposed such as mandatory home confinement and closure of schools, restaurants, all non-essential shops and borders. Outcomes substantially bettered.

All these measures to control the pandemic had significant impact on healthcare and on economic trends in almost all sectors, namely tourism, one of the Portuguese main economic activities. The closure of the borders, and restrictions of travelling to Portugal imposed by other countries, contributed to a rising economic recession.

Previous research has highlighted that economic downturns may have a negative influence on well-being [3], life satisfaction [4], health-related quality of life (HRQol) and mental health [5–6]. Other researchers have shown that changes in unemployment rate had significant impact on well-being [7], and that unemployment or other job-related problems can be seen as determinants for mental health-related difficulties [8]. In addition, in both first and third waves, access to non-emergency and elective care was severely restricted, not only to limit the community-level spread of COVID-19 [1], but also to increase the health system capacity to deal with the rising number of COVID-19 patients. These measures’ uncertain duration may also have contributed to increasing levels of anxiety and decreasing levels of HRQoL and well-being [1].

In conjunction, physical distancing, confinement, economic crisis, restrictions to healthcare access, uncertainty and fear of what would come ahead, fear of being infected or losing a loved one, can have a negative effect on citizens’ mental distress and HRQoL, and a negative impact on citizens’ well-being. Additionally, prohibition of religious celebrations, closure of schools and imposed home-working may also have caused changes on work-balance, thus impacting citizens’ well-being.

Of course, all this scenario of mandatory social restrictions has drastically changed after the vaccination of the whole population, which was very determinant to minimize the risk of death or severe complications from COVID-19 and have helped the entire world to get back to a normal life. In fact, by the end of 2021, Portugal, after a very intense vaccination campaign, has achieved the highest rate of 93.6% of vaccination course completion among all adults [9].

The purpose of this article is to provide evidence on the impact of the COVID-19 on HRQoL and well-being of Portuguese citizens. It aims to (i) characterize these outcomes during the COVID-19 pandemic; (ii) compare them to pre-COVID-19 Portuguese population and with data collected in an early phase of the pandemic; and (iii) identify the social determinants that may have affected these outcomes during the COVID-19 pandemic.

To estimate the impact of COVID-19 on health and well-being of citizens we compared data collected for the purpose of this study (H&W-B COVID survey) with data from previous studies where we have measured pre-COVID HRQoL of the Portuguese population [10], and the HRQoL during the first home quarantine of the population, i.e. during the first lockdown, in an early phase of the pandemic [1].

## Methods

### Sampling design

The target population of this study was 8.7 million Portuguese adults, aged 18 and older [11]. The stratified random sampling method was used to select a representative sample of the population with regards to sex (2 categories), age group (4 groups) and region (7 regions). Furthermore, we have decided to proportionally allocate the global sample size to each stratum. This decision facilitated the estimation of parameters of interest using both point and interval estimation methods, since all sampling units had equal probabilities of selection. The sample size calculation used the population size in each stratum from the Portuguese census 2011 [11] and auxiliary information from the EQ-5D five-level (EQ-5D-5 L) population norms survey [10]. Using the formulae of the stratified sampling with proportional allocation [12: 128], a global sample size of 1,250 individuals was obtained, setting a desired confidence level of 95% and a maximum absolute sampling error of 0.015 to estimate the overall EQ-5D-5 L mean index. At the end, a sample of 1,255 individuals was surveyed, proportional allocated to the 56 strata, using as a criterion for the selection of sampling individuals with the birthday nearest the date of the interview.

The H&W-B COVID survey was performed between 24th March and 20th April 2021, i.e. during the end of the second national lockdown in Portugal, by a market research company. Four trained interviewers collected the data by telephone interview using a Computer Assisted Telephone Interviewing system. Telephone numbers were generated by a computer algorithm to ensure a random sample using a Random Digit Dialing method. The interviews were carried out in about 60% for the mobile phones and the rest for the landlines. The data collection started with mobile phones, randomly distributed throughout the country. Then, interviews were carried out for the landlines in order to comply with the strata by region, age and sex.

Calls were made between 4 p.m. and 10 p.m. when residents were most likely to be available or at home. It was given the possibility of scheduling the interview on another day and/or at a different time for cooperation individuals. It was conducted only one interview per household and each respondent answered to the survey instrument designed for this study after an eligibility check (based on the target population and the sampling design). The telephone interview script was developed by that company and, on average, each telephone call lasted 14 min. The response rate for eligible households contacted by telephone was 35%. A pilot test of the whole survey process was performed in March 2021 by the company. Only after being approved by the research team, through this pilot test, could the company start the fieldwork.

The survey consisted of three parts:

i) General welcome of the participant. After the eligibility check and the selection of the interviewee, it was explained the purpose of the study and each respondent was asked if s/he agreed to participate in the study. Moreover, all respondents were informed that there were no wrong answers, and we assured them about the anonymity and the confidentiality of the data.
ii) Implementation of the survey questions. The theme of all sections of the questionnaire were introduced to respondents before asking the questions, in order the respondent to be contextualized. The scales of measurement were also clearly presented in each question.
iii) General thank you for the cooperation and goodbye. At this stage the interviewees were also informed that they could be contacted again in the future for the purpose of quality control.

The quality control and monitoring of survey was conducted both through direct supervision and third-party phone call listening of 10% of the global sample size. The principles of anonymity, confidentiality and individual privacy were assured along the study. No incentive (monetary or non-monetary) was given to the respondents. This study’s design was reviewed and approved by the University of Algarve’s Research Ethics Committee (ref. CEUAlg 43a/2021).

### Questionnaire

A questionnaire was designed to collect data for the H&W-B COVID survey. The instrument consisted of eight sections to assess citizens’ HRQoL, personal well-being, satisfaction with life, economic and labour impacts, mental and physical health, social networks, access to healthcare and sociodemographic profile.

HRQoL was measured using the EQ-5D-5 L [13]. This instrument is commonly used whether to study the HRQoL of countries’ populations (e.g. [14.–15]), subgroups of the population (e.g. [16].) or patient HRQoL, that can ultimately be also included in health technology assessments. Its descriptive system can be converted into a single index value (EQ-5D-5 L index) ranging from − 0.603 to 1 [17], where 1 is full health and negative values represent states considered worse than death. We have used the Portuguese version of the EQ-5D-5 L applied in [16] and the Portuguese EQ-5D-5 L value set [17] to compute the EQ-5D-5 L index.

To measure the well-being, we have used the Personal Wellbeing Index (PWI) [18]. This PWI scale contains eight items of satisfaction, each one corresponding to a quality of life domain and are theoretically embedded, as representing the first level deconstruction of the global question: ‘How satisfied are you with your life as a whole?’ These items are presented in a 11-points scale and may be summed to yield a score which represents the Subjective Wellbeing (SWB). The Portuguese version of the PWI was used [19].

To measure the satisfaction with life we have used the Satisfaction with Life Scale (SWLS) [20], which is a 5-item scale designed to measure global cognitive judgments of one’s life satisfaction, where higher values mean higher satisfaction. We have used the Portuguese version of the SWLS [21].

To measure the impact of COVID-19 on mental health we have adapted parts of the SHARE Corona Survey [23], aiming to study anxiety, depression, sleeping problems and loneliness before and after the COVID-19 outbreak. To study the impact of COVID-19 pandemic on social networks we have asked the Portuguese citizens on the frequency that they performed usual activities and maintained contacts with family and friends when compared with before the COVID-19 pandemic. These items were also adapted from SHARE Corona Survey [23]. Perceived health both before the COVID-19 outbreak and today was accessed using two items also adapted from SHARE Corona Survey [23]. These items were translated and adapted to Portuguese.

Finally, to measure the perception on economic stability of the household before the pandemic and the maintenance of economic stability we have used a scale by Crosta et al. [22], ranging from 0 (strongly disagree) to 10 (strongly agree). In order to report results in a clearer way, we further created the following categories of economic stability: weak (0–3), fair (4–6) and strong (7–10). Other questions concerning (i) experience of a reduction in salary; (ii) unemployment, lay-off or closure of businesses; (iii) workplace; (iv) postpone regular payments were adapted and translated to Portuguese from SHARE Corona Survey [23].

The sociodemographic profile of the sample was assessed by variables such as sex, age, region of residence, marital status, educational attainment, employment status, household and, religious beliefs.

### Other data for comparison purposes

In order to be able to estimate the impact of COVID-19 on health and well-being of the Portuguese population we have compared H&W-B COVID data with data from previous studies where we have measured pre-COVID HRQoL of the Portuguese population [10], and the HRQoL during the first home quarantine of the population (first lockdown) [1]. The idea was to establish comparisons and to understand how the HRQoL of the PT population was affected by the pandemic.

Data on pre-COVID HRQoL of the Portuguese population came from a national survey that established normative values for the EQ-5D-5 L [13]. A random sample of the Portuguese general population aged 18 or more (n = 1,006) stratified by region, sex and age group was interviewed by trained interviewers between November 2015 and January 2016. This sample matched the Portuguese general population’s characteristics in terms of the stratification variables, but was also similar regarding other variables, such as marital status, employment status, and household. Data was collected via telephone interviews, following a similar study design as was done in the current H&W-B COVID survey. Further details of this study’s methodology can be found elsewhere [10].

Data on HRQoL during the first lockdown came from an online survey conducted between March and April 2020 in Portugal (n = 904) to collect data on anxiety, HRQoL, feelings, duties, and activities performed during the lockdown [1]. The EQ-5D-5 L was used to measure HRQoL. Given that mandatory social distancing and isolation measures were in place at the time of this data collection, the data was collected through online interviews following a combination of convenience and snowball sampling methods to approach the Portuguese population, that was either self-isolated or quarantined [1]. As the final sample did not match the Portuguese population in terms of sex, age and education, the data were weighted according to these variables. Data from the Portuguese census [11] was used to weight the sample and compensate for overrepresentation and underrepresentation of some population groups. At the end, the final sample was very close of the main Portuguese general population’s characteristics. Further details on the sample may be found elsewhere [1].

### Data analysis

Descriptive data analysis was conducted to profile the sample and examine the respondents’ perceptions about the impacts of the COVID-19 on their HRQoL and personal well-being, satisfaction with life, economic and employment status, mental health, social networks, and access to healthcare. Inferential data analysis was also carried out using the EQ-5D-5 L, PWI and SWLS instruments to estimate the study population’s level of HRQoL, personal well-being and satisfaction with life. The EQ-5D-5 L estimatives were compared with the Portuguese population’s norms (i.e. reference values) and with the first lockdown, for which EQ-5D-5 L data were collected in previous cross-sectional studies [1, 10].

Correlations between the EQ-5D-5 L, PWI and SWLS scores were evaluated using Spearman’s rank correlation coefficient. In addition, differences between groups defined by sociodemographic variables were evaluated based on non-parametric tests because those scores do not approximate the normal distribution. The Mann-Whitney U, Kruskal-Wallis H and Pearson’s Chi-square tests were considered to be the most appropriate in this context. A significance level of 5% was used in all statistical tests.

Linear regression models were also used to examine determinants of HRQoL, SWB and satisfaction with life during the COVID-19 lockdown period. This step included investigating the relationships between a set of economic, work, health, and social-related variables included in the questionnaire and the EQ-5D-5 L, PWI and SWLS scores, respectively. The forward stepwise method was applied to select a set of statistically significant explanatory variables in each model. As a result, only statistically significant variables were retained in each model. In addition, diagnosis tests were run in order to examine multicollinearity, autocorrelation and heteroscedasticity assumptions.

## Results

### Study sample

The sample’s main characteristics are shown in Table 1. This profile matches the general population’s characteristics in terms of sex and age. The profile is similar regarding other variables (i.e. marital status, employment status and household). However, the sample has a larger proportion of individuals with a high education attainment (i.e. bachelor’s, master’s or doctoral degree) than the general population does.

**Table 1:** Study sample characteristics and Portuguese general population aged 18 or more

		Sample (*n* = 1,255)	Portuguese general population aged 18 or more* (*N* = 8,657,240)
Sex (%)	Female	51.7	53
	Male	48.3	47.0
Age group (%)	18–29	17.2	17.0
	30–49	35.9	36.3
	50–69	31.6	29.9
	70 +	15.3	16.8
	Mean age (SD)	48.8 (17.5)	49.1 (18.5)
Marital status (%)	Single	24.9	27.4
	Married/living with a partner	61.4	56.9
	Divorced/separated	8.0	6.9
	Widowed	5.7	8.9
Educational attainment (%)	Low	30.5	62.1
	Medium (secondary)	35.0	19.1
	High (bachelor, master, doctorate)	34.5	18.8
Employment status (%)	Employed	58.8	50.1
	Unemployed	7.6	7.4
	Retired	24.7	27.0
	Student	4.6	4.6
	Domestic	2.7	4.8
	Another situation	1.6	6.1
Household (%)	1 to 2 elements	41.9	53.0
	3 to 4 elements	48.8	40.5
	5 or more elements	9.3	6.5
Residence (%)	City	50.8	n.a.
	Small town	20.0	n.a.
	Small village	29.1	n.a.
COVID-19 risk group in household (%)	Yes	40.5	n.a.
	No	59.5	n.a.
Religious belief (%)	Yes	81.3	n.a.
	No	18.7	n.a.

* Source: Census 2011 [20]

Note: n = number; SD = standard deviation; n.a. – not available

The majority of the respondents were female (51.7%). Almost 36% of the respondents were aged between 30 and 49 years old, followed by those aged 50–69 years old (31.6%). Overall, the respondents’ mean age was 48.8 years old. The majority were married or living with a partner (61.4%) and one fourth were single (24.9%).

Close to one third of the respondents (30.5%) indicated they had a low level of education; the percentage of respondents with a medium or high education level is almost the same (35.0% /34.5%). In addition, 58.8% reported that they were employed, 24.7% were retired, and 7.6% were unemployed. Close to half of respondents lived in a household with 3–4 members (48.8%), followed by those living in households with 1–2 members (41.9%). More than a half lived in a city (50.8%), followed by those living a small village (23.3%). About 40.5% of respondents belonged to or lived with someone from the COVID-19 risk group, i.e., people aged 65 years and older and with underlying health conditions, such as hypertension, diabetes, cardiovascular disease, chronic respiratory disease and/or weakened immune systems. Finally, the majority (81.3%) reported a belief in the existence of a God or a higher being.

### Health and access to healthcare

Respondents were asked to describe their perceived health before the pandemic in the H&W-B COVID survey. They were also asked if their health had worsened, stayed the same or improved during the pandemic. Only one-fourth of the Portuguese citizens perceived their health as very good before the pandemic, and almost half reported a good health. In addition, only 4% of the citizens perceive their health as bad or very bad before the pandemic. We also estimated that 79.0% of citizens consider their health the same and, 18.0% worse during the pandemic than before the pandemic. The remaining 3.0% perceived their health as better.

Table 2 relates perceived health during the pandemic with perceived health before the pandemic. Results show that there is a greater percentage of citizens feeling worse during the pandemic (22.0%) that reported a reasonable health before the pandemic when compared with those that reported a very good (15.0%) or a good (18.0%) health. In addition, 21.0% of citizens that perceived their health as bad before the pandemic feel better during the pandemic, while the percentage of citizens that feel better during the pandemic is lower or equal to 5% in the remaining pre-COVID perceived health categories. There is significant evidence that perceived health during the pandemic is dependent of the perceived health before the pandemic (χ^2^ = 46.2; *p*-value < 0.001).

**Table 2:** Comparison of perceived health before and during the pandemic (%)

Perceived health before the pandemic	Perceived health during the pandemic		
	Worse	The same	Better
**Very good**	15	82	3
**Good**	18	80	2
**Reasonable**	22	74	4
**Bad**	20	59	21
**Very bad**	14	86	0

In addition, we also wanted to know whether the respondents or someone that lived with him/her have had COVID-19. Our findings show that 17.5% of the respondents or someone that lived with them had had COVID-19. From these, 12.7% needed to stay in the hospital due to complications related to COVID-19 and from those who had to stay in the hospital, almost half (46.4%) had to stay in an intensive care unit.

Moreover, considering the overall share of citizens reporting surgeries, medical appointments or complementary exams postponed or cancelled, and the distribution by age group, we evidence that the access to healthcare by Portuguese citizens and patients has decreased during the pandemic, not only in surgeries postponed or cancelled, but also in medical appointments across all specialties over the course of the pandemic and on complementary exams. In fact, from our study, we estimate that whilst 4.2% of citizens had their surgeries postponed or cancelled because of COVID-19, more than a quarter had medical appointments or complementary exams postponed or cancelled. Table S1 of the Supplementary Material reports the percentage of citizens with surgeries, medical appointments or complementary exams postponed or cancelled because of COVID-19.

Analysing now the access to healthcare by age group, we conclude that access to healthcare by citizens and patients is significantly dependent of the age group (medical appointments or complementary exams: χ^2^ = 11.9; *p*-value = 0.008; surgeries: χ^2^ = 6.7; *p*-value = 0.084). Same table S1 shows that the oldest groups had a greater number of postponements/cancellations than younger: 7.3% of citizens over 70 years old had surgeries postponed or cancelled and 31.4% had medical appointments or complementary exams postponed or cancelled.

### Health-related quality of life and mental health

Table 3 presents the relative frequency distribution by EQ-5D-5 L dimensions. Results presented show that COVID-19 pandemic significantly impacted negatively the HRQoL of citizens, especially in the first lockdown (H = 80.3; p-value < 0.001). This impact was somehow mitigated in the second lockdown, denoting that citizens seem to have learned how to cope with the pandemic. We estimate that during the first lockdown their HRQoL decreased 2.9%, and in 2021 it has increased, even attaining levels higher than before the pandemic (+ 1,2%).

**Table 3:** Distribution of relative frequencies by EQ-5D-5 L dimensions (%)

Dimension	Level	Pre-COVID ^a^	1st lockdown (March-April 2020) ^b^	2nd lockdown (March-April 2021)
Mobility	No problems Slight problems Moderate problems Severe problems Extreme problems	74.9 11.6 9.8 3.1 0.6	82.0 7.3 3.8 4.2 2.7	78.2 11.6 8.0 1.9 0.3
Self-care	No problems Slight problems Moderate problems Severe problems Extreme problems	91.2 4.3 3.9 0.3 0.3	85.2 7.5 1.6 3.0 2.7	93.9 2.9 2.4 0.4 0.3
Usual activities	No problems Slight problems Moderate problems Severe problems Extreme problems	75.9 10.5 10.0 2.8 0.8	70.4 15.3 10.6 0.6 3.1	77.2 14.7 6.1 1.4 0.5
Pain/discomfort	No problems Slight problems Moderate problems Severe problems Extreme problems	45.9 34.2 15.3 4.0 0.6	62.3 24.5 10.0 3.2 0.0	52.9 31.2 13.1 2.8 0.1
Anxiety/depression	No problems Slight problems Moderate problems Severe problems Extreme problems	60.9 24.4 11.3 2.0 1.4	40.7 37.6 14.2 0.9 6.6	48.9 31.3 16.6 2.4 0.8
EQ-5D-5 L index	Mean (SE)	0.887 (0.005)	0.861 (0.027)	0.898 (0.004)
	Median (IQ)	0.959 (0.137)	0.956 (0.103)	0.956 (0.132)

SE-Standard error. IQ-Inter-Quartile range

^a^ Data from the EQ-5D-5 L Portuguese population norms [10]

^b^ Data from the 1st lockdown [1]

Table S2 of the Supplementary Material also reports the EQ-5D-5 L index by sociodemographic characteristics. Results show that HRQoL varied by sociodemographic groups in the second lockdown. In fact, there is significant evidence that HRQoL is different by sex (U = 152,479; *p*-value < 0.001) and age group (H = 103.5; *p*-value < 0.001). Men report better HRQoL than women, while younger groups report better HRQoL than older groups. Statistically significant declines in EQ-5D-5 L index means were observed in almost all categories of the variables considered. Comparing the EQ-5D-5 L index in 2021 with the period pre-pandemic and to the first lockdown, it is possible to see that the pandemic impact was stronger in the first lockdown across all sociodemographic characteristics.

In addition, Table 4 shows that half of the Portuguese citizens felt nervous, anxious, or on edge and for 85.0% of citizens, these feelings were more so than before the COVID-19 pandemic. Almost half of Portuguese citizens felt sad or depressed and 85.0% felt more so than before the pandemic. Sleeping problems were reported for 39.0% of citizens and 71.0% had these problems more so than before the pandemic. Finally, loneliness was reported by 32.0% of citizens (26.0% some of the time and 6.0% often), and these feelings have increased with the COVID-19 pandemic.

**Table 4:** Distribution of frequency of feelings of anxiety, sadness, depression, sleeping problems, loneliness and of performance of social activities (%)

Feelings	Last month			When compared with pre-COVID		
	No	Yes		More so	About the same	Less so
Feeling nervous, anxious, or on edge	50	50		85	12	3
Feeling sad or depressed	55	45		85	12	3
Trouble sleeping	61	39		71	26	3
	**Hardly ever or never**	**Some of the time**	**Often**	**More so**	**About the same**	**Less so**
Feeling lonely	68	26	6	75.0	21.0	4.0
**Social activities**	**I never realized**	**Less frequently**		**More or less the same**	**More frequently**	**Not applicable**
Going shopping	3	59		34	3	1
Going for a walk	11	43		31	13	2
Meeting socially with more than 5 people outside their household	58	35		5	1	1
Visiting other family members	38	54		7	0	1

### Social activities

Table 4 also presents the results on the frequency of social activities, such as going shopping, going for a walk, visiting other family members, or meeting socially with people outside their household. We estimated that the majority of Portuguese citizens went shopping less frequently than before or stopped shopping (62.0%) and that they went for a walk also less frequently or stopped walking (54.0%). Almost 60% of citizens reported that they no longer meet more than five people outside their household since the outbreak of COVID-19, and 35% reported that they met less frequently. Visits to other family members were also less frequent for the majority of citizens and more than one third stopped visiting since the outbreak of COVID-19.

### Work and economic situation

The H&W-B COVID survey intended to understand, as well, the perception of Portuguese citizens on their economic stability before the pandemic. The majority of the Portuguese citizens (86.0%) perceived that they had a strong economic stability before the pandemic, while 13.0% perceived it as fair and only 1.0% as weak. Using a scale ranging from 0 (strongly disagree) to 10 (strongly agree), we estimate that the level of agreement of the citizens with their economic stability before the pandemic is 8.4.

To determine if the perceived economic stability has changed due to the pandemic, we analysed the level of agreement of the citizens with a maintenance of their economic stability in the last year. Results show that there is significantly evidence that agreement with the maintenance of the citizens’ economic stability increases with economic stability before the pandemic (H = 122.6; *p*-value < 0.001). In addition, there is also a significant and positive correlation between economic stability of the household before and during the pandemic (ρ = 0.506; *p*-value < 0.001). Data also shows that the level of agreement with a maintenance of economic stability in the last year in the group of citizens with a strong economic stability before the pandemic is 7.7, while in the small group of citizens with a weak economic stability before the pandemic is 4.9.

In the H&W-B COVID survey, citizens were also asked whether they experienced a reduction in salary, a job loss or permanent business closure or had to postpone regular payments such as rent or loans due to the pandemic. Results are shown in Supplementary Material (table S3). More than one-third of Portuguese citizens experienced a reduction in salary or turnover (35.5%), 14.3% lost their job or had to permanently close their business and 10% had to postpone regular payments due to the pandemic.

Our findings also highlight that the pandemic did not impact in the same way citizens from different age groups, since a loss of job or permanent closure of the business (χ^2^ = 255.3; *p*-value < 0.001), a reduction in salary or the business turnover (χ^2^ = 274.6; *p*-value < 0.001), and a postponement of regular payments (χ^2^ = 65.9; *p*-value < 0.001) are significantly dependent of the age group. Whilst less than 15.0% of active age citizens lost their jobs or had to close their companies (table S3 of the Supplementary Material), this percentage rises to almost a quarter in the case of citizens aged 18–29. We also evidenced that almost half of citizens aged 18–29 and of citizens aged 35–49 experienced a reduction in income. It also illustrates that about 16.0% of citizens aged 30–49 had to postpone regular payments, such as rent or loans, due to the COVID-19 pandemic, which is also significantly higher than in older age groups (50–69 and 70+).

### Satisfaction with life and well-being

**Fig. 1:**
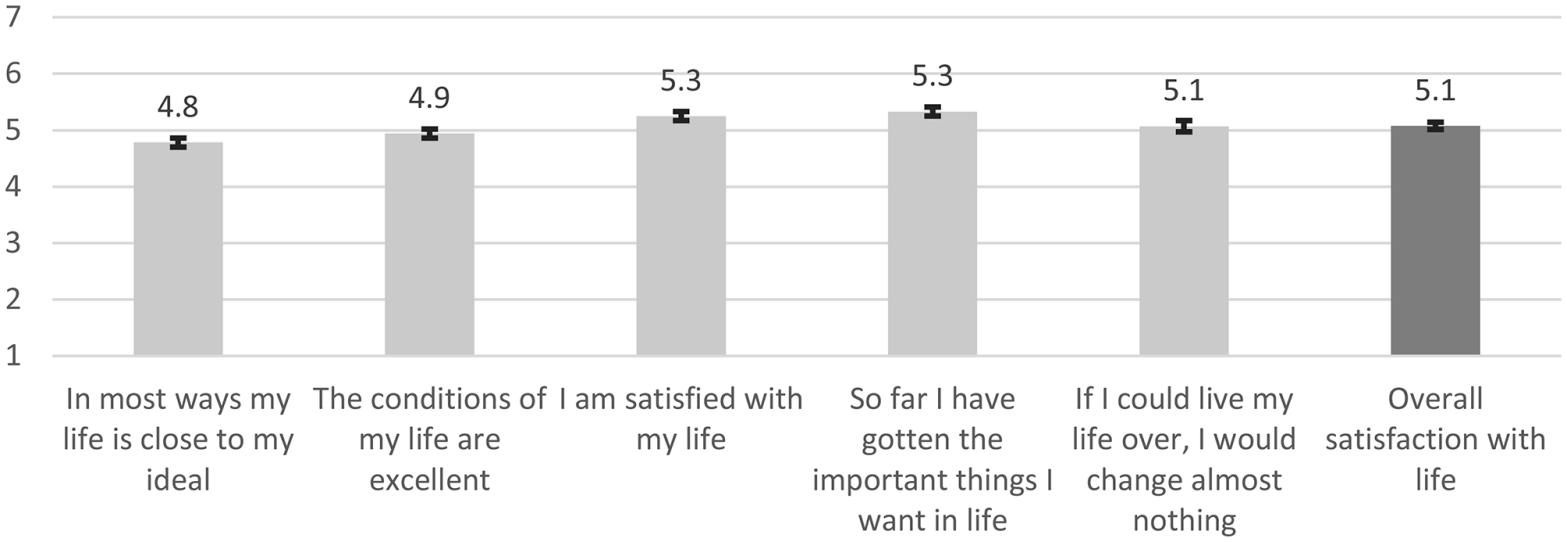
Satisfaction with life

The H&W-B COVID survey intended to access Portuguese citizens’ satisfaction with life. When surveyed in March-April 2021, Portuguese citizens were asked, on a scale of 1 to 7, about their level of agreement with five items that measure satisfaction with life, i.e. the SWLS. Results presented in Fig. 1 show that Portuguese citizens are generally satisfied with life, since the estimated average overall satisfaction with life is 5.1 points (1–7 scale). Overall, the estimated SWLS global score sum 25.4 points, meaning that the Portuguese citizens are slightly satisfied with life.

**Fig. 2:**
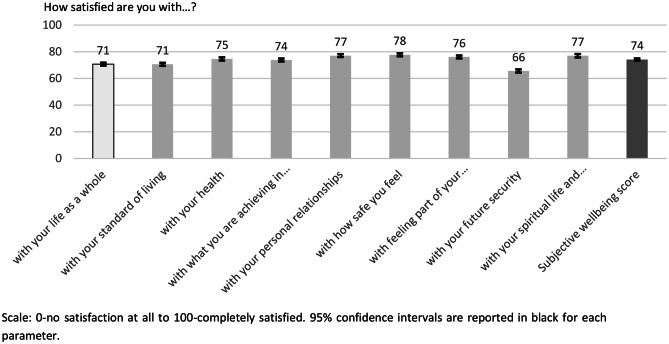
Subjective well-being

Figure 2 presents the results of the SWB, which has been obtained from the PWI. Portuguese citizens reported a moderate well-being, since the estimated average of SWB is 74, within the normative range of 70 to 80 for Western countries [18]. Regarding the eight items of satisfaction, citizens report values that range from 66 to 78, although the majority are above 70. The highest level of satisfaction of the citizens is with how safe they feel (78), while the lowest level of satisfaction is with their future security (66). It is also important to note that the estimated average score for satisfaction with life as a whole of citizens is 71, which shows their capacity to cope with the impacts of pandemic.

Three linear regression models were estimated used to identify the determinants of HRQoL, SWB and satisfaction with life during the COVID-19 lockdown period. Results presented in Table 5 show that the coefficients of determination of these models range from 0.196 to 0.291 and all models are statistically significant (*p*-value_F−statistic_<0.001). In what concerns the robustness tests, first of all, the variance inflation factors (VIF) indicated that there were two variables with a VIF exceeding the potential problematic level of 10. That led to merging these two dummy variables into a single dummy (feeling lonely often or some of the time), which solved the multicollinearity problem. The null hypothesis of the Breusch-Pagan test was rejected in the first models estimated, indicating that there was a problem with heteroscedasticity. In order to solve this problem, coefficients were estimated using robust standard errors in the reported models. Finally, the Durbin-Watson statistics indicate that autocorrelation is not present in the residuals of all models (ranging from 1.938 to 2.093), as expected since we are using cross-sectional data.

**Table 5:** Estimates of models for SWB, Satisfaction with life and HRQoL

Independent variables	SWB		Satisfaction with life		HRQoL	
	Beta	CI of Beta	Beta	CI of Beta	Beta	CI of Beta
Having an appointment or complementary exam postponed or cancelled because of COVID-19	-0.333***	[-0.528 ; -0.138]			-0.037***	[-0.056 ; -0.018]
Having to postpone regular payments, such as rent or loans because of COVID-19	-0.480**	[-0.767 ; -0.192]	-2.613***	[-3.864 ; -1.363]		
Having experienced a job loss or permanent business closure because of COVID-19			-1.545**	[-2.561 ; -0.528]		
Feeling nervous, anxious, or on edge	-0.289**	[-0.490 ; -0.089]	-0.786*	[-1.570 ; -0.002]	-0.015*	[-0.029 ; -0.001]
Feeling sad or depressed	-0.351**	[-0.560 ; -0.141]	-1.453***	[-2.295 ; -0.611]	-0.045***	[-0.061 ; -0.028]
Having trouble sleeping	-0.345***	[-0.548 ; -0.143]	-1.468***	[-2.236 ; -0.700]	-0.048***	[-0.064 ; -0.031]
Feeling lonely often or some of the time	-0.484***	[-0.675 ; -0.293]	-1.794***	[-2.503 ; -1.085]	-0.025**	[-0.043 ; -0.007]
Living in the same home with someone belonging to a covid-19 high-risk group	-0.273**	[-0.448 ; -0.099]			-0.032*	[-0.047 ; -0.016]
Going shopping frequently					0.016*	[ 0.002 ; 0.030]
Going out for a walk frequently					0.014**	[ 0.005 ; 0.024]
R-square	0.221		0.196		0.291	

SWB-subjective well-being; HRQoL – Health-related quality of life; CI – 95% Confidence Interval. ***p < 0.001. **p < 0.01. *p < 0.05

Table 5 also shows the estimated unstandardized regression coefficients, their two-tailed *p*-values, and 95% confidence intervals for the estimated coefficients. Our findings show that the following variables have a negative statistically significant effect on SWB of the Portuguese citizens: have an appointment or complementary exam postponed or cancelled because of COVID-19; have to postpone regular payments, such as rent or loans, because of COVID-19; feel nervous, anxious, or on edge; feel sad or depressed; have troubles to sleep; feel lonely often or some of the time; and live in the same home with someone belonging to the risk group of COVID-19. For example, the estimated coefficient − 0.289 means that feeling nervous, anxious or on edge is associated with a 0.289 decrease in SWB, after controlling for other variables.

Following the same methodological approach, results reported in Table 5 revealed that the following variables are significantly associated with satisfaction with life of the Portuguese citizens in a negative way: have to postpone regular payments, such as rent or loans, because of COVID-19; have experienced a loss of job or permanently closure of the business because of COVID-19; feel nervous, anxious, or on edge; feel sad or depressed; have troubles in sleeping; and feel lonely often or some of the time. For example, individuals that have had to postpone regular payments reported a lower level of satisfaction with life (-2.613) than other.

Finally, Table 5 shows that the following variables negatively explain HRQoL of the Portuguese citizens: have an appointment or complementary exam postponed or cancelled because of COVID-19; feel nervous, anxious, or on edge; feel sad or depressed; have troubles in sleeping; feel lonely often or some of the time; and live in the same home with someone belonging to the risk group of COVID-19. In addition, this regression model also showed that there are also variables significantly affecting HRQoL in a positive way: to go shopping and to go out for a walk frequently. This means that these two variables are associated with a slight rise (0.016 and 0.014, respectively) in HRQoL, *ceteris paribus*.

## Discussion

Our study presents findings on how the Portuguese population was affected by the pandemic and the imposed measures to fight the pandemic. It is based on a national survey that aimed at studying the impact of the pandemic in a broad way, analysing health and access to healthcare, mental health, HRQoL, satisfaction with life and well-being. Our study comprehends not only HRQoL results measured during the second national lockdown, but also from before the pandemic and during the first lockdown, i.e., in an early phase of the pandemic.

The results of this study show that health of Portuguese citizens has deteriorated since the start of the pandemic. The results also show that perceived health during the pandemic is dependent from the perceived health before the pandemic (e.g. almost one fourth of citizens feeling worse during the pandemic reported a reasonable health before the pandemic). These results are similar to Dziedzic and colleagues’ findings [23], that reported a deterioration in physical health during the pandemic among the elderly in Poland. TurSinai and colleagues [24] also found similar results, since they reported a worsening of health relative to pre-pandemic in a vast majority of European and Israeli older adults following the first wave of the COVID-19 pandemic.

There is clear evidence that access to healthcare by citizens and patients has decreased during the pandemic all over the world [25] and this was also the case for Portugal, where hospitals have reduced elective surgery aiming at protecting patients from in-hospital viral transmission, releasing critical care beds for surgeries in COVID-19 patients and repurposing recovery areas to intensive care units. Our results also show that medical appointments or complementary exams have also been extensively postponed or cancelled, in line with the worldwide decrease in healthcare access (e.g. [25–27].). In addition, Valente de Almeida et al. [28] showed that planned medical appointments were postponed or converted into remote appointments during the first emergency state in Portugal.

Additionally, our findings suggest that this decrease is associated with citizens’ age group since the reduction to access to healthcare was more intense for older citizens during the pandemic of COVID-19, as has been reported by others (e.g. [29.–30]).

COVID-19 pandemic significantly impacted negatively the HRQoL of citizens, especially in the first lockdown and this negative impact in HRQoL has been reported by others (e.g. [31–34].). Nevertheless, this impact was somehow mitigated in the second lockdown. In fact, the Portuguese citizens reported a slightly higher level of HRQoL during the second lockdown than before the pandemic. These results may be justified, not only by the ability of the populations to cope with extreme situations, but also with the increase of vaccination rate, speeded-up by mid-March 2021, or with the NHS ability to deal with the increasing needs of the Portuguese citizens.

In this study, the mean EQ-5D-5 L index varied significantly by age, sex, education, marital status, employment status, and place of residence. The age variation (decreasing HRQoL with increased age) was in accordance with other studies (e.g. [31, 35].), as well as the gender differences in terms of impact, with several studies reporting a lower HRQoL in women’s HRQoL and a stronger negative impact on women’s health assessment compared to men (e.g. [31, 34, 36].). Similar effects were also found for education, since higher levels are associated with better HRQoL (e.g. [31, 37].) and for employment status, where unemployed, retired/pensioners and homemakers report lower levels of HRQoL in the second lockdown (e.g. [31, 38].), but higher values than at the early beginning of the pandemic.

Half of the Portuguese citizens felt nervous, anxious, or on edge, and a high percentage felt sad or depressed and reported sleeping problems. These problems increased during the pandemic. Our results are in accordance with Cohn-Schwartz et al. [39], that found high levels of loneliness between European adults during the pandemic. Other studies also found prevalence of increased depression/sadness during the pandemic, increased anxiety and other mental problems (e.g. [23, 31, 40].).

Furthermore, Portuguese citizens reported that their social networks were severely affected by the pandemic. In fact, more than half of Portuguese citizens did not meet more than five people outside their household during the pandemic. These results are similar to those of Cohn-Schwartz et al. [39] that reported high levels of physical distancing for European adults. Their results also showed that these adults felt lonelier during the pandemic, suggesting that even though physical distancing guidelines can decrease the risk of COVID-19 infection, they may contribute to the increase of loneliness.

In addition, our results show that the majority of the Portuguese citizens perceive that they had a strong economic stability before the pandemic. This study also highlights that economic stability of Portuguese citizens weakened during the pandemic of COVID-19 and shows that there is significantly evidence that agreement with the maintenance of the citizens’ economic stability increases with economic stability before the pandemic. These findings are in accordance with those of Andrade et al. [41] that showed that the pandemic has had a significant impact on finances and relationships. In addition, it has also been shown that this financial stress may impact both couple and family well-being and relationships in a variety of ways [42, 43].

It has been reported that the pandemic may be portrayed as a stressful situation in the face of the major disruptions in family and working lives, with impacts on the emotional and physical well-being of the citizens (e.g. [41, 44, 45].). COVID-19 and the associated lockdowns and changes in lifestyle have increased stress, and even conflict, within relationships, have cause financial stress, reduction in healthcare access, and many other changes that impact life satisfaction and well-being. Our findings have added to the existing research on the area and helped to identify which factors are more likely to explain life satisfaction and well-being. In fact, our results show that feelings of loneliness, sadness, nervousness, anxiety and trouble sleeping had a negative effect in life satisfaction, well-being and HRQoL. Appointments or health exams cancelled or postponed had also a negative effect in well-being and HRQoL. Finally, financial problems are also negatively associated with satisfaction with life and well-being. On the contrary, the maintenance of social contact had a positive effect in HRQoL. Our results are in line with those of Ammar et al. [46], that reported a close relationship between social distancing and life dissatisfaction, revealing that social contact with family and friends was negatively affected by home confinement and that it is important to stay in touch to keep an acceptable level of life satisfaction.

Despite all the consequences of the pandemic that affected the different populations, the Portuguese citizens at the time of the survey (i.e. second lockdown) were slightly satisfied with life and reported a moderate well-being, showing that at the time of the survey the Portuguese citizens have learned to cope with the pandemic. In addition, at the time of the survey the vaccination was already in course and this has probably led to the moderate well-being results; Portuguese citizens were probably very hopeful on the success of the vaccination.

### Strengths and limitations

Our study has several strengths. First, we surveyed a national representative sample of Portuguese citizens in variety of different areas, such as health and access to health, mental health, HRQoL, working and economic situation, social networks, satisfaction with life and well-being. There are few studies, to the best of our knowledge, that have included such a variety of variables in a national sample. Moreover, we have used existing validated instruments to measure satisfaction with life, well-being and HRQoL. In addition, we were able to compare data on HRQoL with existing data collected before the pandemic and in an early phase of the pandemic.

However, this study has also some limitations. In fact, although having three samples and being able to perform comparisons with data pre-pandemic and with data collected during and in an early phase of the pandemic may be seen as a strength, it can also be seen as a limitation. First, the data collection modes were different (two samples were phoned interviewed while one sample was contacted electronically), and this may have been associated to a non-measurable interview bias. Second, the disruptions caused by the pandemic could have impacted the final results, since it is not possible to fully understand how much were differences in findings between the surveys (e.g., pre-COVID and COVID) the result of changes in experiences and quality of life brought on by the lockdowns or pandemic versus the differences in the separate survey samples.

Another limitation is related to the time of the survey. Data was collected at the end of second lockdown and when the vaccination was already in course. When the survey was designed, the vaccination was still very incipient and this was the case for not considering variables related to the vaccination - there were very few people vaccinated in Portugal at that time. Nevertheless, results on satisfaction with life and well-being may have been affected by the hope that the population may have at that time in vaccines, and this cannot be disentangled.

Another limitation is related to not being able to establish comparisons regarding satisfaction with life and well-being as was done with HRQoL, i.e., compare our results with data collected before the pandemic and with data collected at the first lockdown, to fully understand the impact of the pandemic.

## Conclusion

Overall, this study suggests that Portuguese citizens—especially older ones— experienced limitations in assessment to healthcare during the pandemic, and reported worse mental health and interruptions in their social networks. In terms of economic situation, people with the greatest stability before the pandemic were more likely to report that their situation had been maintained during the pandemic. Despite the challenges of the pandemic, the study reveals that Portuguese citizens were slightly satisfied with life and reported a moderate well-being during the second lockdown. In addition, the study identifies the determinants that affected Portuguese citizens’ subjective wellbeing, life satisfaction and HRQoL.

These findings can help health authorities and policymakers to prepare the health system to be able to cope with citizens’ healthcare needs during emergencies and to develop psychological interventions to ensure citizens’ mental health and thus better HRQoL and well-being. Regarding loneliness and other mental issues associated with loneliness, while there is a potential for social relations to be maintained via technology-based solutions, remote social contacts cannot fully compensate for the loss of physical contacts [47]. Therefore, policy responses to futures pandemics or other crisis need to consider these issues to avoid unnecessary increases in loneliness, sadness, anxiety and depression. Moreover, this study sheds light on actions that need to be taken to help Portuguese citizens recover their social networks and improve their economic situation.

### Electronic supplementary material

Below is the link to the electronic supplementary material.

Supplementary Material 1

## Data Availability

The data that support the findings of this study are available on the website of Social Observatory’s of “la Caixa” Foundation upon request or can be requested from the corresponding author.
