# Supplementary material for: Health and well-being of the Portuguese citizens: impacts of the COVID-19
Source: J Patient Rep Outcomes. 2023 Sep 5;7:88. doi: 10.1186/s41687-023-00628-1 (PMC10480107; doi:10.1186/s41687-023-00628-1)
Supplement: Supplementary file 1 — Supplementary Material 1 [file 41687_2023_628_MOESM1_ESM.docx]

Table S1 - Citizens with surgeries, medical appointments or complementary exams postponed or cancelled (%)

|  |  | **Age group** | | | |
| --- | --- | --- | --- | --- | --- |
|  | **Total** | **18-29** | **30-49** | **50-69** | **70 +** |
| Appointment or complementary exam postponed or cancelled because of COVID-19 | 28.3 | 21.9 | 25.6 | 33.3 | 31.4 |
| Surgery postponed or cancelled because of COVID-19 | 4.2 | 2.3 | 3.8 | 4.3 | 7.3 |

Table S2. EQ-5D-5L index by sociodemographic characteristics

| **Characteristic** |  | **Pre-COVID ^a^** | | **1^st^ lockdown**  **Mar-Apr 2020 ^b^** | | **2^nd^ lockdown Mar-Apr 2021** | |
| --- | --- | --- | --- | --- | --- | --- | --- |
|  |  | **Mean** | **SE** | **Mean** | **SE** | **Mean** | **SE** |
| Sex | Male  Female | 0.914  0.863 | 0.007  0.007 | 0.943  0.789 | 0.013  0.050 | 0.919  0.878 | 0.005  0.006 |
| Age group | 18 – 29  30 – 49  50 – 69  70 + | 0.962  0.919  0.868  0.790 | 0.005  0.007  0.010  0.016 | 0.916  0.845  0.894  0.600 | 0.007  0.044  0.028  0.268 | 0.945  0.928  0.865  0.844 | 0.005  0.005  0.008  0.013 |
| Education | Low  Medium  High | 0.848  0.912  0.930 | 0.009  0.009  0.007 | 0.826  0.911  0.929 | 0.044  0.006  0.005 | 0.844  0.923  0.921 | 0.010  0.005  0.005 |
| Marital status | Single  Married/living with a partner  Divorced/separated  Widowed | 0.927  0.889  0.865  0.774 | 0.009  0.006  0.020  0.029 | 0.797  0.888  0.826  0.925 | 0.076  0.026  0.061  0.016 | 0.929  0.900  0.842  0.818 | 0.005  0.005  0.018  0.024 |
| Employment status | Employed/self-employed  Unemployed  Retired/pensioner  Student  Homemaker | 0.928  0.875  0.812  0.962  0.845 | 0.005  0.017  0.014  0.011  0.023 | 0.885  0.839  0.814  0.912  0.742 | 0.027  0.005  0.093  0.008  0.131 | 0.927  0.891  0.837  0.935  0.881 | 0.004  0.016  0.010  0.010  0.019 |
| Place of residence | City  Small town  Small village | 0.906  0.894  0.860 | 0.007  0.010  0.011 | 0.857  0.900  0.852 | 0.041  0.019  0.052 | 0.909  0.898  0.880 | 0.005  0.008  0.009 |
| Household size | 1-2  3-4  5+ | 0.856  0.907  0.927 | 0.009  0.008  0.015 | 0.858  0.859  0.919 | 0.035  0.048  0.014 | 0.868  0.923  0.905 | 0.007  0.004  0.015 |
| Religious beliefs | Yes  No | n.a.  n.a. | n.a.  n.a. | 0.847  0.906 | 0.037  0.025 | 0.888  0.928 | 0.005  0.007 |
| Living with a family member at risk | Yes  No | n.a.  n.a. | n.a.  n.a. | 0.729  0.895 | 0.093  0.019 | 0.866  0.921 | 0.007  0.004 |

SE-Standard error. n.a.-not available.

^a^ Data from the EQ-5D-5L Portuguese population norms[10].; ^b^ Data from the 1^st^ lockdown[1].

Comparisons of the EQ-5D-5L index score distributions by variables 1, 8 and 9 were analyzed using the Mann-Whitney U test. All other differences amongst groups were analyzed with the Kruskal-Wallis H test.

Table S3 - Citizens that lost a job or closed their business, suffered a reduction in salary or turnover, or postponed regular payments due to the pandemic (%)

|  |  | **Age group** | | | |
| --- | --- | --- | --- | --- | --- |
|  | **Total** | **18-29** | **30-49** | **50-69** | **70 +** |
| Job loss or permanent business closure | 14.3 | 23.3 | 17.1 | 11.9 | 2.6 |
| Reduction in salary or turnover | 35.5 | 44.5 | 47.1 | 30.4 | 10.5 |
| Postpone regular payments such as rent or loans | 10.0 | 9.8 | 15.3 | 7.6 | 2.6 |
